# Screening of a library of traditional Chinese medicines to identify anti-malarial compounds and extracts

**DOI:** 10.1186/s12936-018-2392-4

**Published:** 2018-06-25

**Authors:** Motohiro Nonaka, Yuho Murata, Ryo Takano, Yongmei Han, Md. Hazzaz Bin Kabir, Kentaro Kato

**Affiliations:** National Research Center for Protozoan Diseases, Obihiro University of Agriculture and Veterinary Medicine, Inada-cho, Obihiro, Hokkaido 080-8555 Japan

**Keywords:** Antimalarial drugs, Drug screening, Traditional Chinese medicine, *Plasmodium falciparum*

## Abstract

**Background:**

Malaria is a major infectious disease in the world. In 2015, approximately 212 million people were infected and 429,000 people were killed by this disease. *Plasmodium falciparum*, which causes falciparum malaria, is becoming resistant to artemisinin (ART) in Southeast Asia; therefore, new anti-malarial drugs are urgently needed. Some excellent anti-malarial drugs, such as quinine or ART, were originally obtained from natural plants. Hence, the authors screened a natural product library comprising traditional Chinese medicines (TCMs) to identify compounds/extracts with anti-malarial effects.

**Methods:**

The authors performed three assays: a malaria growth inhibition assay (GIA), a cytotoxicity assay, and a malaria stage-specific GIA. The malaria GIA revealed the anti-malarial ability and half-maximal inhibitory concentrations (IC_50_) of the natural products, whereas the malaria stage-specific GIA revealed the point in the malaria life cycle where the products exerted their anti-malarial effects. The toxicity of the products to the host cells was evaluated with the cytotoxicity assay.

**Results:**

Four natural compounds (berberine chloride, coptisine chloride, palmatine chloride, and dehydrocorydaline nitrate) showed strong anti-malarial effects (IC_50_ < 50 nM), and low cytotoxicity (cell viability > 90%) using *P. falciparum* 3D7 strain. Two natural extracts (*Phellodendri cortex* and *Coptidis rhizoma*) also showed strong antiplasmodial effects (IC_50_ < 1 µg/ml), and low cytotoxicity (cell viability > 80%). These natural products also demonstrated anti-malarial capability during the trophozoite and schizont stages of the malaria life cycle.

**Conclusions:**

The authors identified four compounds (berberine chloride, coptisine chloride, palmatine chloride, and dehydrocorydaline nitrate) and two extracts (*Phellodendri cortex* and *Coptidis rhizoma*) with anti-malarial activity, neither of which had previously been described. The IC_50_ values of the compounds were comparable to that of chloroquine and better than that of pyrimethamine. These compounds and extracts derived from TCMs thus show promise as potential future anti-malarial drugs.

## Background

Malaria is major, global infectious disease. In 2015, approximately 212 million people were infected and 429,000 people were killed by this infection, mainly in tropical and subtropical regions [1]. Malaria is caused by various *Plasmodium* protozoal parasites (*Plasmodium falciparum*, *Plasmodium vivax*, *Plasmodium malariae*, *Plasmodium ovale*, *Plasmodium knowlesi*) [2]; falciparum malaria, caused by *P. falciparum,* is responsible for most of the malaria-related deaths globally and severe cases of malaria [1].

The main drug currently used to treat malaria is artemisinin (ART). Its derivatives are used in artemisinin-based combination therapy (ACT) and have helped countless patients all over the world [3]. However, *P. falciparum* is becoming resistant to ART in Southeast Asia. When resistance to chloroquine (CQ) and sulfadoxine (SD)-pyrimethamine (PYR) spread from Southeast Asia and South America to Africa, many African people, especially children, died. To avoid a similar outcome due to ART resistance, the authors must develop new anti-malarial treatment options [4].

Humans have used plants for medicine since ancient times. Indeed, the first anti-malarial drugs, quinine (QN) and quinidine (QND), were found in the bark of the Cinchona tree and have been used for over 350 years as an anti-malarial drug in South America [5]. In China, traditional Chinese medicines (TCMs) have a more than 2000-year history and their appropriate use has been developed over a long time [6]. ART, which has become one of the best known anti-malarial drugs, was also discovered from a plant, *Artemisia annua*. This plant is a famous TCM identified by Dr. Tu who established screening protocols of Chinese traditional herbs and was awarded the Nobel Prize in Physiology or Medicine in 2015 [3].

Clearly, some excellent anti-malarial drugs have been derived from natural plants. Here, the authors were fortunate to obtain a natural product library comprising TCMs. This library contained 96 different compounds and 120 different extracts that had never been evaluated for anti-malarial activity. Many of compounds were refined from the extracts. Hence, the authors screened this natural product library with the aim of identifying compounds and extracts with anti-malarial activities. The authors found several hit compounds and extracts that could be candidates for new drug against malaria parasites.

## Methods

### Compounds

The natural drug library was provided by the Institute of Natural Medicine (The University of Toyama, Toyama, Japan). Pyrimethamine (Wako, Osaka, Japan) and artesunate (ATN; Sigma, MO, USA) were also used for the screening, as described below.

### *Plasmodium falciparum* culture in vitro

The authors used *P. falciparum* 3D7 and Dd2 strain blood stage parasites for this study. Parasites were grown in AB+ human red blood cells (RBCs) and maintained in culture medium containing RPMI 1640, 25 mM HEPES, 100 µM hypoxanthine, 12.5 µg/ml gentamycin, 0.5% (w/v) Albumax II, and 62.5 µg/ml NaHCO_3_. The culture was maintained at 37 °C, 5% O_2_, and 5% CO_2_, with daily medium changes.

### Cell culture

Human embryonic kidney derived (HEK) 293T cells were grown in culture medium containing DMEM, 10% fetal bovine serum (FBS), l-glutamine, penicillin–streptomycin, and 62.5 µg/ml NaHCO_3_ at 37 °C, with 5% O_2_ and 5% CO_2_. The cells were passaged every 2 days at 70–80% confluency.

### *Plasmodium falciparum* growth inhibition assays (GIAs)

GIAs were performed as previously described [7]. Briefly, cultures containing mainly ring-stage parasites were synchronized by means of d-sorbitol treatment. After 36 h, cultures containing mostly ring-stage parasites growing up again were again synchronized by use of sorbitol treatment. GIAs were initiated on the next day when the synchronized parasites grew up to the late trophozoite stage. Infected human red blood cells (iRBCs) were mixed with fresh type AB+ uninfected human RBCs (uRBCs) to prepare cultures with 0.3% parasitaemia and 1% haematocrit. All compounds were dissolved in DMSO at the concentration of 10 mM, and all extracts were dissolved in distilled water at 100 mg/ml. The authors prepared each required concentration of compounds and extracts in serial dilution methods. The cultures were then transferred into 96-well plates at 147 µl per well, and 3 µl of compounds/extracts were added to each well. All experiments were done in triplicate. The plates were incubated at 37 °C, with 5% O_2_ and 5% CO_2_. At 48 h post-incubation, 50 µl of complete medium was added to each well. The degree of inhibition was assessed by determining the parasitaemia by using an optical microscope at 96 h post-incubation when the parasites were mostly at the trophozoite or schizont stages. The authors determined the growth inhibitory rate as follows, growth inhibitory rate (%) = [1 − {(parasitaemia of sample) − (parasitaemia of positive control)}/{(parasitaemia of negative control) − (parasitaemia of positive control)}] × 100. IC_50_ was determined by analysis of dose–response curve made by GraphPad Prism (GraphPad Software, CA, USA).

### Cytotoxicity assays

Cell Counting Kit-8 (Dojindo, Kumamoto, Japan) was used for the cytotoxicity assays. HEK 293T cells were cultured using 96-well microplates in 100 µl of medium for 96 h with various concentrations of hit compounds (10^1^, 1, 10^−1^, 10^−2^, 10^−3^ µM) or extracts (10^2^, 10^1^, 1, 10^−1^, 10^−2^, 10^−3^ µg/ml). After the 96-h incubation, 10 µl of Cell Counting Kit solution was added to each well and incubated for 3 h. Then, the absorbance at 450 nm was measured using a plate reader (Corona Electric, Ibaraki, Japan). The authors determined the cell viability as follows, cell viability (%) = {(absorbance at 450 nm of treated group)/(absorbance at 450 nm of control group)} × 100. Parasite selectivity is important index to evaluate the practical use of the compounds and extracts for malaria treatment; high numbers have high parasite selectivities. The authors also calculated the parasite selectivity as follows, parasite selectivity (%) = {(IC_50_ value of host cell)/(IC_50_ value of parasite)} × 100.

### Stage-specific growth inhibition assays

Stage-specific GIAs were carried out similarly to standard GIAs [8], but stage specificity was initiated immediately after the second synchronization. iRBCs were mixed with fresh type AB+ uRBCs to prepare cultures with 0.3% parasitaemia and 1% haematocrit. Cultures were transferred into a 96-well plate at 147 µl per well. Cultures were divided into five groups: ring-stage samples, trophozoite-stage samples, schizont-stage samples, positive control (10 µM PYR), and negative control (0.1% DMSO), and 3 µl of compounds/extracts were added simultaneously to each well, first for 0–24 h into the ring-stage wells, next for 24–36 h into the trophozoite wells, and last for 36–48 h into the schizont wells. After exposure to the compounds/extracts, the RBCs were washed three times with culture medium, and cultured until 48 h post-incubation. These experiments were performed in triplicate. The cultures were incubated at 37 °C, with 5% O_2_ and 5% CO_2_. The amount of compounds/extracts was assessed by determining the parasitaemia of Giemsa-stained samples by using an optical microscope at 48 h post-incubation when the parasites were mostly in the trophozoite or schizont stages.

## Results

### Screening of the natural compound library for anti-Plasmodium effects

**Fig. 1:**
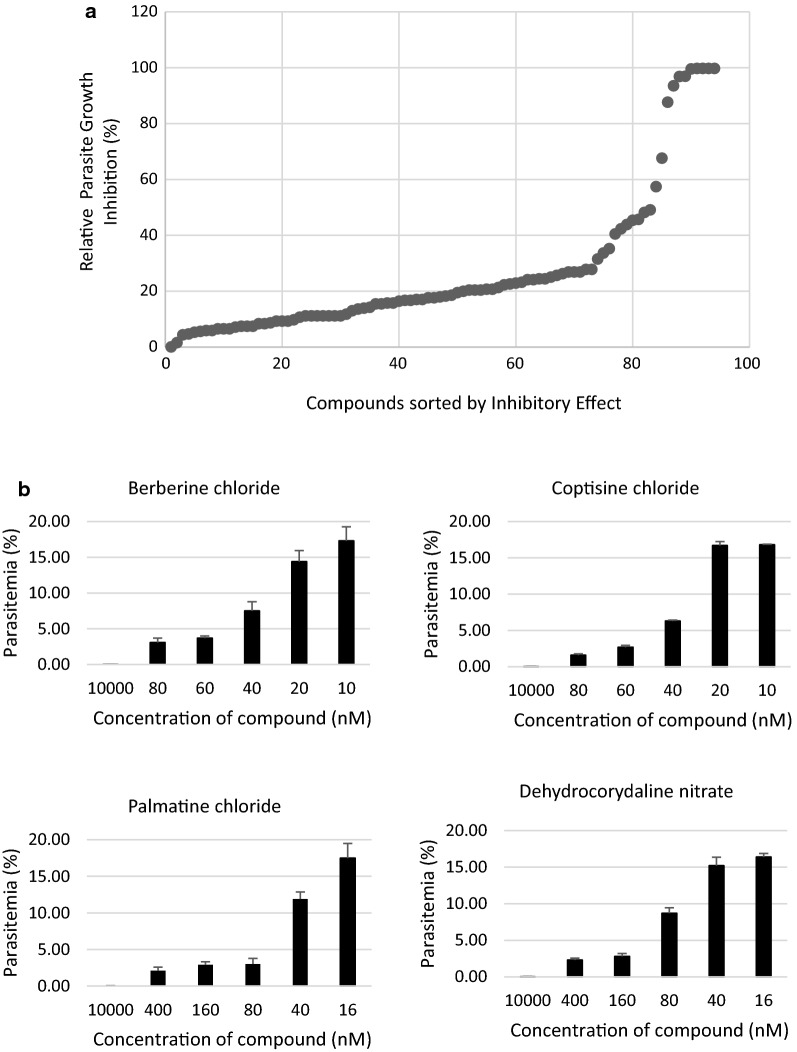
Natural compound screening for anti-malarial activity using 3D7 strain. **a** *Plasmodium falciparum* 3D7 strain-infected human RBCs were incubated with 10 µM test compounds (96 different compounds) for 96 h (see “Methods”). Five compounds showed almost 100% growth inhibitory effects. **b** The four selected compounds (berberine chloride, coptisine chloride, palmatine chloride, and dehydrocorydaline nitrate) were investigated for concentration-dependent growth inhibition effects. Six concentrations of each compound were incubated with parasites for 96 h. Concentration-dependent anti-malarial effects were observed and IC_50_ values were calculated (see Table 1)

**Fig. 2:**
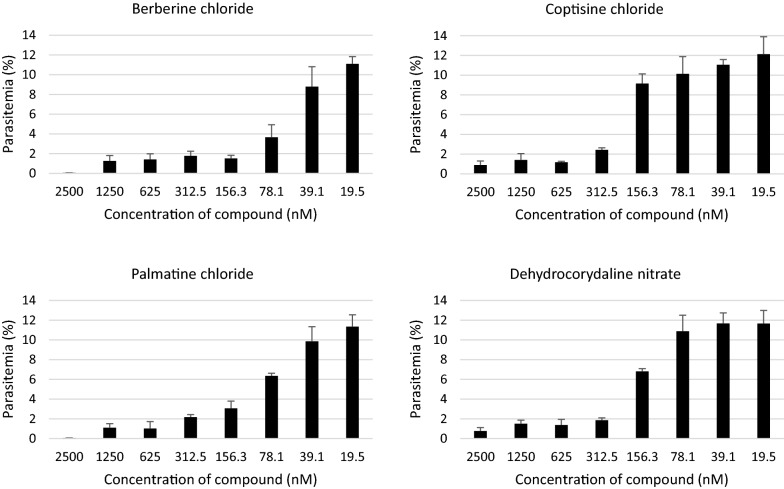
Natural compound screening for anti-malarial activity using Dd2 strain. The four hit compounds were investigated for concentration-dependent growth inhibitory effects. Eight concentrations of each compound were incubated with parasites for 96 h. Concentration-dependent growth inhibition were observed and IC_50_ values were calculated (see Table 2)

The authors screened 96 natural compounds at a final concentration of 10 µM to assess their *P. falciparum* growth inhibitory effects. Parasitaemia in the wells to which 0.1% DMSO was added was set as 0% growth inhibition, whereas that in the wells treated with 10 µM PYR, which is enough to inhibit the growth of *P. falciparum* 3D7 strain, was calculated as almost 100% inhibition. Of these 96 compounds, five (berberine chloride, coptisine chloride, palmatine chloride, dehydrocorydaline nitrate, and timosaponin A-III) inhibited growth by > 99% (Fig. 1a). This is the first report of anti-malarial activity for dehydrocorydaline nitrate. Since timosaponin A-III had a haemolytic effect, the authors selected the other four compounds (Table 1) for further assessment. Next, the authors determined the half maximal inhibitory concentrations (IC_50_) of the four compounds (Fig. 1b); the IC_50_ values of all four compounds were < 50 nM (Table 1). Additionally, the authors assessed anti-malarial effect of the four compounds using *P. falciparum* Dd2 strain. 10 µM ATN, which is enough to inhibit the growth of *P. falciparum* Dd2 strain, was used as the positive control. The four compounds inhibited Dd2 strain parasite growth similarly (Fig. 2), and IC_50_ values were < 200 nM; higher than 3D7 strain (Table 2). Previously reported IC_50_ values for CQ and PYR were 27.1 nM and 733.26 nM, respectively [9, 10], indicating that the IC_50_ values of the authors’ test compounds were comparable to or better than those of CQ and PYR.

**Table 1:** Hit compounds from the first screening using *P. falciparum* 3D7 strain

Compound	Growth inhibition rate (%) IC_50_ (SEM) (nM)
Palmatine chloride	99.5 26 (24–28)
Berberine chloride	99.7 36 (34–38)
Coptisine chloride	99.7 35 (34–36)
Dehydrocorydaline nitrate	99.7 38 (36–39)
Timosaponin A-III	99.7 Not determined due to its haemolytic effect

**Table 2:** Hit compounds screening using *P. falciparum* Dd2 strain

Compound	Growth inhibitory rate (%) IC_50_ (SEM) (µg/ml)
Berberine chloride	100 54 (50–59)
Palmatine chloride	100 81 (75–87)
Dehydrocorydaline nitrate	94.5 161 (149–174)
Coptisine chloride	93.5 190 (172–210)

Berberine chloride, coptisine chloride, and palmatine chloride are isoquinoline alkaloids, some of which have previously been shown to have antiplasmodial activity [11]. The authors’ data indicate that a dehydrocorydaline nitrate, which has a similar structure to that of an alkaloid, can be added to the list of isoquinoline alkaloid-like compounds with inhibitory activity against plasmodial parasites.

### Screening of the natural extract library for anti-*Plasmodium falciparum* effects

**Fig. 3:**
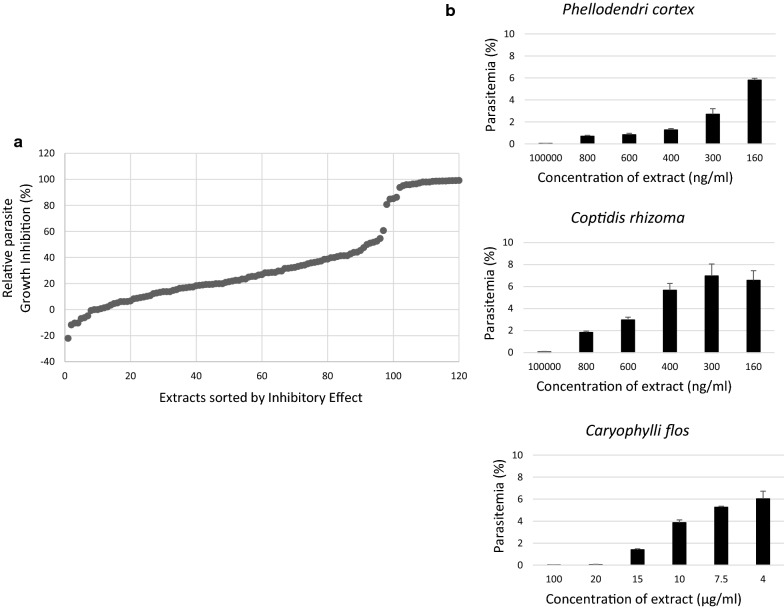
Natural extract screening for anti-malarial activity using 3D7 strain. **a** *Plasmodium falciparum* 3D7 strain-infected human RBCs were incubated with 100 µg/ml test extracts (120 different extracts) for 96 h (see “Methods”). Nineteen extracts showed > 90% growth inhibitory effects. **b** Three selected extracts (*Caryophylli flos*, *Coptidis rhizoma*, and *Phellodendri cortex*) were investigated further for concentration-dependent growth inhibition effects. Different concentrations of each extract were incubated with parasites for 96 h. Concentration-dependent anti-malarial effects were observed and IC_50_ values were calculated (see Table 2)

**Fig. 4:**
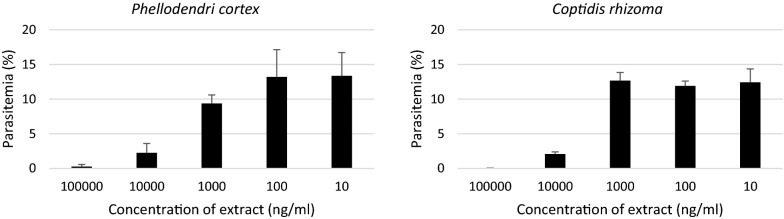
Natural extract screening for anti-malarial activity using Dd2 strain. The two hit extracts were investigated for concentration-dependent growth inhibitory effects. Five concentrations of each extract were incubated with parasites for 96 h. Concentration-dependent growth inhibition were observed and IC_50_ values were calculated (see Table 4)

The authors screened 120 natural extracts at a final concentration of 100 µg/ml for *P. falciparum* 3D7 strain growth inhibitory effects. Parasitaemia in the wells to which only sterilized distilled water was added was set as 0% growth inhibition, whereas that in the wells treated with 10 µM PYR was calculated as 100% inhibition. Of the 120 extracts screened, 19 inhibited growth by > 90% (Fig. 3a). The authors chose the nine extracts with the greatest inhibitory effects from these 19 and determined their IC_50_ values. Three of the nine (*Phellodendri cortex*, *Coptidis rhizoma*, and *Caryophylli flos*) showed strong anti-malarial effects at 20 µg/ml (Fig. 3b), and the IC_50_ values of two of them (*Phellodendri cortex* and *Coptidis rhizoma*) were < 1 µg/ml (Table 3). This is the first-time anti-malarial activity for these three extracts has been reported. Also, the authors determined the anti-malarial effect of the two extracts using *P. falciparum* Dd2 strain. Positive control was 10 µM ATN. Dd2 strain parasite growth was inhibited by the extracts similarly (Fig. 4), and IC_50_ values were higher than those of 3D7 strain (Table 4).

**Table 3:** Hit extracts from the first screening using *P. falciparum* 3D7 strain

Extract	Growth inhibition rate (%) IC_50_ (SEM) (µg/ml)
*Forsythiae fructus*	98.6 > 20
*Myrrha*	98.8 > 20
*Rhei rhizoma*	98.8 > 20
*Quercus cortex*	99.0 > 20
*Spatholobi caulis*	99.0 >20
*Caryophylli flos*	98.6 11.5 (11.1–11.8)
*Coptidis rhizoma*	99.1 633 × 10^−3^ (601–665)
*Phellodendri cortex*	99.2 298 × 10^−3^ (285–310)

**Table 4:** Hit extracts screening using *P. falciparum* Dd2 strain

Extract	Growth inhibition rate (%) IC_50_ (SEM) (µg/ml)
*Coptidis rhizoma*	100 827 × 10^−3^ (463–1474)
*Phellodendri cortex*	98.8 651 × 10^−3^ (384–1107)

### Cell viability

**Fig. 5:**
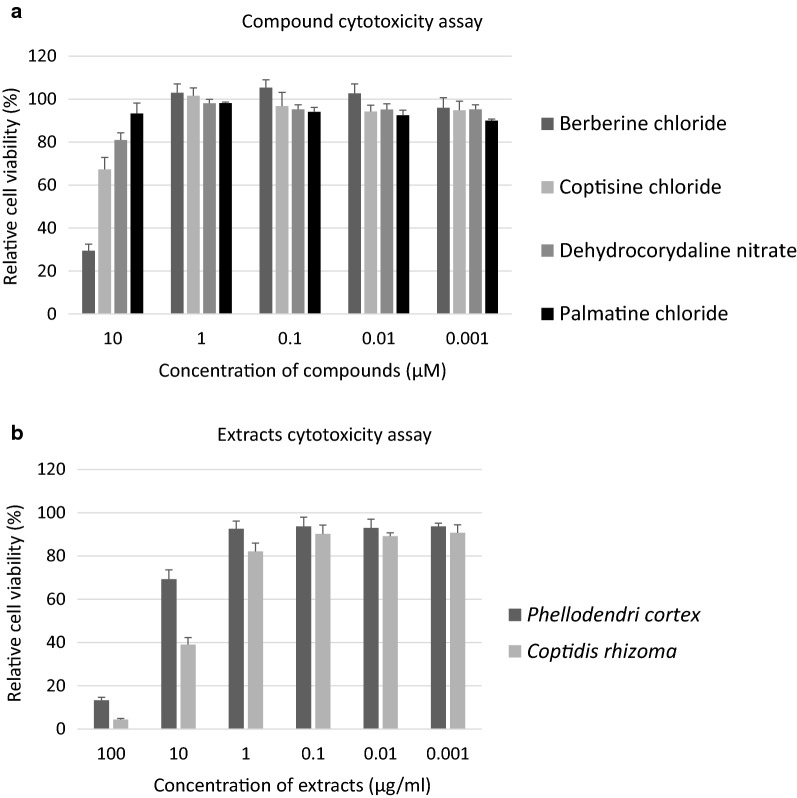
Cytotoxicity of the natural compounds and extracts. **a** Cytotoxicity assays were performed with the four anti-malarial compounds. 293T cells were incubated with different concentrations of test compounds for 96 h (see “Methods”). The authors observed high cell viability in the presence of all four compounds at concentrations of < 1 µM. **b** Cytotoxicity assays were performed with the two anti-malarial extracts. 293T cells were incubated with various concentrations of the test extracts for 96 h (see “Methods”). The authors observed high cell viability in the presence of both extracts at concentrations of < 1 µg/ml

The effects of the four compounds and two extracts on the viability of HEK 293T cells was assessed. Absorbance at 450 nm of wells containing 0.1% DMSO or sterilized distilled water was set as 100% cell viability. Cell viability in the presence of any of the four compounds was > 90% and in the presence of either extract was > 80% (Fig. 5a, b). In addition, the authors calculated the *P. falciparum* selectivity of these compounds and extracts, and found that all four compounds had > 100 selectivity and that the two extracts had ≤ 10 selectivity (Table 5).

**Table 5:** Parasite selectivity of hit compounds and extracts

Compounds and extracts	Selectivity (%)
Palmatine chloride	> 45,500
Coptisine chloride	> 29,400
Berberine chloride	24,200
Dehydrocorydaline nitrate	> 14,900
*Phellodendri cortex*	11,000
*Coptidis rhizoma*	1000
*Caryophylli flos*	300

The results suggest that these compounds and extracts may be safe as anti-malarial drugs because they showed strong effects against only parasites and had little effect on human cells.

#### Stage-specific growth inhibition

**Fig. 6:**
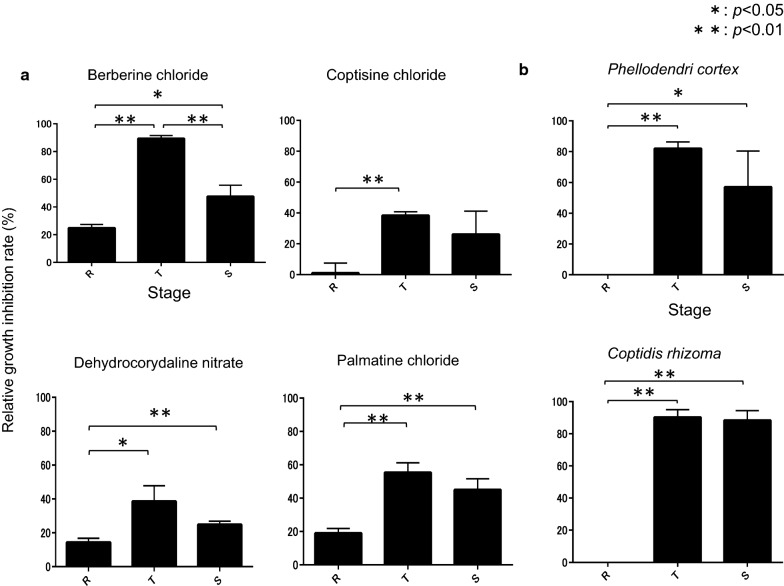
Stage-specific growth inhibition by the compounds and extracts. **a** *Plasmodium falciparum* 3D7 strain-infected human RBCs were incubated with 10 µM test compounds during each stage (see “Methods”). Significant anti-malarial effects during the ring-form, trophozoite, and schizont stages were observed. **b** *P. falciparum* 3D7 strain-infected human RBCs were incubated with 100 µg/ml test extracts during each stage (see “Methods”). Significant anti-malarial effects during the ring-form, trophozoite, and schizont stages were observed

Stage-specific growth inhibition activities of the compounds and extracts were also investigated using *P. falciparum* 3D7 strain. Berberine chloride, dehydrocorydaline nitrate, and palmatine chloride showed growth inhibition effects during the trophozoite and schizont stages (Fig. 6a). Berberine chloride showed a particularly strong effect during the trophozoite stage. Coptisine chloride showed an effect during the trophozoite stage, but may also exhibit an effect during the schizont stage (Fig. 6a). *Phellodendri cortex* and *Coptidis rhizoma* also showed effects during the trophozoite and schizont stages (Fig. 6b), and their effects were stronger than those of the natural compounds. The authors observed growth inhibitory effects during the trophozoite and schizont stages with both the compounds and extracts.

## Discussion

Here the authors identified compounds and extracts with anti-malarial activity from a library of TCMs. Anti-malarial activity for berberine has been reported previously, but its intensity differed from that obtained in this study. In the authors’ investigation, the IC_50_ of berberine chloride was 31 nM; however, other studies reported IC_50_ values of 270 nM (using FCR3 strain, cultured for 72 h) [12], < 100 nM (using 3D7 strain, cultured for 72 h) [13], 80 nM (using K39 strain, cultured for 66 h), and 1.98 µM (using V1/S strain, cultured for 66 h) [14]. In these studies, different strains of *P. falciparum* were used and the culture time was also shorter than the authors’. The FCR3 and V1/S strains have multi-drug resistance (FCR3 is resistant to CQ and cycloguanil; V1/S is resistant to CQ, SD, and PYR). In contrast, the 3D7 and K39 strains are not multi-drug resistant (3D7 is resistant to SD; K39 is resistant to PYR). Growth of Dd2 strain (resistance to CQ and mefloquine) was inhibited at a little higher drug concentration, suggesting that difference in parasite strains did not much contribute the difference in IC_50_ values. With respect to culture time, the authors’ experiments were performed for 96 h, whereas the others were performed within 72 h. This means that the authors’ parasites were exposed to the natural products twice, because the life cycle of malaria is 48 h, but only once in these other studies. Therefore, the authors believe that these differences in IC_50_ values resulted from the differences in the culture times used.

The authors’ study showed that berberine chloride, coptisine chloride, palmatine chloride, and dehydrocorydaline nitrate have strong anti-malarial activity; their base compounds (berberine, coptisine, palmatine, and dehydrocorydaline) belong to the isoquinoline alkaloids. Berberine and its analogues, which include dehydrocorydaline, are called protoberberine alkaloids and their anti-malarial activity has been reported previously [11, 12, 15–17]. However, anti-malarial activity of dehydrocorydaline nitrate has not been previously demonstrated even though it is a protoberberine alkaloid. The authors’ study is thus the first to reveal this activity of dehydrocorydaline nitrate. This result also highlights the possibility for discovery of other as yet unrevealed natural products that could contribute to overcoming malaria disease. Because the molecular structures of coptisine and palmatine are similar to that of berberine, these two compounds can be regarded as members of the protoberberine alkaloids. Thus, protoberberine alkaloids may have any common sites or moiety that performs the anti-malarial effect observed.

Few studies have analysed natural extracts for anti-malarial activity. *Phellodendri cortex*, *Coptidis rhizoma*, and *Caryophylli flos* have not been directly evaluated in such studies even though they have been used as a source of extracts such as berberine. The authors’ study is the first to investigate the anti-malarial activity of the three extracts, and the authors discovered that they do indeed have growth inhibitory effects on malaria parasites. In particular, *Phellodendri cortex* has a low IC_50_ value and high selectivity, suggesting it may be of value in developing a novel strategy against malaria. Indeed, *Phellodendri cortex* includes berberine and palmatine, and *Coptidis rhizoma* includes berberine, palmatine, and coptisine. Therefore, the activity of these two natural extracts may be caused by the isoquinoline alkaloids they contain. In contrast, the other six extracts of the eight the authors studied in detail do not include isoquinoline alkaloids. Moreover, one component of *Quercus cortex* is an uncharacterized entity. Therefore, the authors may discover additional novel anti-malarial compounds from TCMs in the future.

The authors cultured *P. falciparum* 3D7 strain parasites in culture medium with 10 times the IC_50_ value of berberine chloride for 3 months in an attempt to produce a berberine-resistant malaria parasite, but the authors were unable to obtain such a parasite. A previous study reported that berberine inhibits the telomerase activity of *P. falciparum* K1 strain [18, 19]. Telomeres play important roles in eukaryotic cells in maintaining genome stability and ensuring accurate DNA replication. It may be difficult to maintain cells without these functions, so the fact that the authors were unable to create the resistant parasite suggests that berberine and other analogues may be instrumental in the development of novel anti-malarial drugs because these compounds inhibit the telomerase activity of *P. falciparum* but not host cells at low concentration (Table 5).

Extracts showed stronger anti-malarial effect than compounds during trophozoite and schizont stages. This difference may be caused by the difference between a single compound and a complex extract, in other words, the power of the extract complex was stronger than that of the individual compounds. Few studies have evaluated the stage-specific anti-malarial effects of natural products; therefore, the authors’ study may help elucidate the mechanism of the inhibitory effect. The authors observed growth inhibitory effects during the trophozoite and schizont stages with both the compounds and extracts. The authors’ result suggests that these natural products use an inhibitory mechanism that suppresses a development process of the *plasmodium* parasite, such as gene expression or DNA replication.

## Conclusions

The authors identified four compounds (berberine chloride, coptisine chloride, palmatine chloride, and dehydrocorydaline nitrate) and two extracts (*Phellodendri cortex* and *Coptidis rhizoma*) that show anti-malarial activity by screening a library of TCMs. The authors’ study is the first to determine that dehydrocorydaline nitrate, *Phellodendri cortex*, and *Coptidis rhizoma* can inhibit the growth of both 3D7 and Dd2 strains of malaria parasites. The IC_50_ values obtained for these compounds were equivalent to that of CQ [9] and better than that of PYR [10]. The authors’ findings indicate that these compounds and extracts derived from TCMs may be of value in the development of new anti-malarial drugs.

## Abbreviations

ACT: artemisinin-based combination therapy
TCM: traditional Chinese medicine
RBC: red blood cell
GIA: growth inhibition assay
IC_50_: half maximum inhibitory concentration
ART: artemisinin
ATN: artesunate
QN: quinine
QND: quinidine
CQ: chloroquine
SD: sulfadoxine
PYR: pyrimethamine
